# Characterization of Romanian Bee Pollen—An Important Nutritional Source

**DOI:** 10.3390/foods11172633

**Published:** 2022-08-30

**Authors:** Mircea Oroian, Florina Dranca, Florin Ursachi

**Affiliations:** Faculty of Food Engineering, Stefan cel Mare University of Suceava, 720229 Suceava, Romania

**Keywords:** bee pollen, physicochemical parameters, fatty acids, amino acids

## Abstract

Bee pollen represents an important bee product, which is produced by mixing flower pollens with nectar honey and bee’s salivary substances. It represents an important source of phenolic compounds which can have great importance for importance for prophylaxis of diseases, particularly to prevent cardiovascular and neurodegenerative disorders, those having direct correlation with oxidative damage. The aim of this study was to characterize 24 bee pollen samples in terms of physicochemical parameters, organic acids, total phenolic content, total flavonoid content, individual phenolics compounds, fatty acids, and amino acids from the Nort East region of Romania, which have not been studied until now. The bee pollen can be considered as a high protein source (the mean concentration was 22.31% d.m.) with a high energy value (390.66 kcal/100 g). The total phenolic content ranged between 4.64 and 17.93 mg GAE/g, while the total flavonoid content ranged between 4.90 and 20.45 mg QE/g. The high protein content was observed in *Robinia pseudoacacia*, the high content of lipids was observed in *Robinia pseudoacacia* pollen, the high fructose content in *Prunus* spp. pollen while the high F/G ratio was observed in *Pinaceae* spp. pollen. The high TPC was observed in *Prunus* spp. pollen, the high TFC was observed in *Robinia pseudoacacia* pollen, the high free amino acid content was observed in *Pinaceae* spp. pollen, and the high content of PUFA was reported in *Taraxacum* spp. pollen. A total of 16 amino acids (eight essential and eight non-essential amino acids) were quantified in the bee pollen samples analyzed. The total content of the amino acids determined for the bee pollen samples varied between 11.31 µg/mg and 45.99 µg/mg. Our results can indicate that the bee pollen is a rich source of protein, fatty acids, amino acids and bioactive compounds.

## 1. Introduction

Bee pollen is collected by honeybees (*Apis* spp.) and is stored and used as food for all development stages in the hive [1]. Bee pollen is a honey bee derivate that is produced by mixing flower pollens with nectar (and/or) honey and bee’s salivary substances. The main compounds found in the bee pollen are: proteins (10–40% in dry weight), carbohydrates (13–55% in dry weight), lipids (1–13% in dry weight), dietary fibers (0.3–20% in dry weight), phenolic compounds (up to 2.5% in dry weight), fatty acids, minerals, amino acids, carotenoids and vitamins [2]. The phenolic compounds (e.g., flavonoids and phenolic acids) presented in the bee pollen are of a great interest for pharmaceutical industry due to their great importance for prophylaxis of diseases, particularly to prevent cardiovascular and neurodegenerative disorders, those having direct correlation with oxidative damage [3]. Given its unique composition, bee pollen is consumed as a food supplement and scientists considered that it is an important functional food [2,4,5] and was reported to have strong health properties such as antioxidant, antiallergen, anti-inflammatory, antiulcer, immune-stimulating, antimicrobial and anticarcinogenic [6,7]. The chemical composition of bee pollen is influenced by different factors such as: floral source, geographical origin, and harvesting technique [8,9,10]. Carbohydrates represent 13–55% in dry weight of the bee pollen depending on botanical and geographical origin of the product; the main carbohydrates present are fructose, glucose and sucrose (more than 90% of the total carbohydrates content) [11]. Proteins represent a high percentage of bee pollen (10–40% in dry weight), but the amino acids define much better the biological value of the bee pollen; they play an important role in human nutrition (e.g., in metabolism, reduce excessive body fat, modulates gene expression, enhances skeletal muscle) [12,13,14,15]. The essential amino acids presented in the bee pollen represent 34.59% to 48.49% of the total content of amino acids; the main amino acids presented are aspartic acids, leucine, glutamic acid, proline and lysine [16]. The bee pollen was reported to have a high antioxidant activity mainly due to the polyphenols which generate a high free radical scavenging potential [10,14,17,18,19,20]. Among the phenolic compounds reported to be determined in the bee pollen were: kaempferol, caffeic acid, quercetin, isoquercetrin, galangin and chrysisn; the glycosides of isorhamnetin, quercetin and kaempferol are the predominant flavonoids in bee pollen [3,21,22]. Bee pollen is a rich source of oil (1–13% in dry weight) and in consequence an important source of fatty acids for hive development; they are important not only for their role as a structural component for cell membranes and energy, but also for their role for bees health [12,23,24,25,26,27]. The main fatty acids presented in the bee pollen are saturated fatty acids (e.g., myristic acid (C14:0), palmitic acid (C16:0), and stearic acid (C18:0)) followed by unsaturated fatty acids (e.g., oleic acid (C18:1), linoleic acid (C18:2), and alpha-linolenic acid (C18:3)) [28]. There were reported two polyunsaturated fatty acids (linoleic acid (omega-6 fatty acid) and alpha-linolenic acid (omega-3 fatty acid)) in the bee pollen, which cannot be biosynthesized by humans and bees [25].

In the last year there has been a high interest from humans and the scientific community for using natural sources as alternatives for synthetic drugs, however the knowledge regarding the bee pollen from Romania is little, and the consumption of it is not high. To gather the two demands, it is necessary to classify the pollen according to the botanical origin and to deeply characterize it from physicochemical point of view in order to achieve its characteristics. Bee pollen can be used as a food supplement due to its positive effects as an antioxidant or antimicrobial effects; moreover, the low content of sugars and saturated fatty acids make the bee pollen a perfect component for food diets [7,29]. The composition of bee pollen is correlated to the botanical origin, geographical origin (the climatic and pedological factors influence the chemical composition) and the processing techniques used [29,30].

Nowadays, the bee pollen is considered a functional food product due to its high nutritional value and chemical composition (e.g., vitamins, carotenoids, phenolic compounds) [5]. The bee pollen extracts can be used for complementary treatment of different diseases (e.g., benign prostatic hyperplasia, vasomotor symptoms), but the most important compounds that may pose a high pharmacological activity are phenolic acids, fatty acids, flavonoids, carotenoids [31]. In the market, the bee pollen can be found as capsules, granules, pellets and powders [32], and the daily recommended dose for an adult is from 20 to 40 g [5].

The aim of this study is to characterize 24 samples of bee pollen in terms of physicochemical parameters (pH, free acidity, protein content, oil content, moisture content), fatty acids, amino acids, carbohydrates, organic acids, individual phenolics compounds and FT-IR spectra. Until now there have been no other studies related to the characterization of bee pollen samples from the North-East part of Romania.

## 2. Materials and Methods

### 2.1. Bee Pollen Samples

A total of 24 samples of bee pollen from the North East part of Romania were collected in 2020 from spring to autumn, and the samples were dried. The samples were kept at −20 °C prior to analysis.

### 2.2. Materials

H_2_SO_4_ (37%), NaOH (>99%), metaphosphoric acid (>99%), gluconic acid (>99%), formic acid (>99%), butyric acid propionic acid (>99%), lactic acid(>99%), acetic acid (100%), rosmarinic acid (>99%), *p*-coumaric acid(>99%), chlorogenic acid (>99%), vanillic acid (>99%), caffeic acid (>99%), *p*-hydroxybenzoic acid (>99%), protocatechiuc acid (>99%), gallic acid (>99%), kaempferol (>99%), quercetin (>95%), luteolin (>99%), myricetin (>99%), methanol (>99%), fructose (>99%), glucose (>99%), sucrose (>99%), melesitose (>99%), raffinose (>99%), AlCl3 (>99%), sodium carbonate (>99%), trichloroacetic acid (>99%) were purchased from Sigma Aldrich (Germany). Fatty acids methyl esters (FAME) mix was purchased from Restek, (Bellefonte, PA, USA, 35077). Alanine, sarcosine, glycine, α-aminobutyric acid, valine, β-amino isobutyric acid, internal standard, leucine, allo-isoleucine, isoleucine, threonine, serine, proline, asparagine, thioproline, aspartic acid, methionine, 3-hydroxyproline/4-hydroxyproline, phenylalanine, glutamic acid, α-aminoadipic acid, α-aminopimelic acid, glutamine, ornithine, glycyl-prolizine—2 isomers, proline-hydroxyproline, histidine, lysine, tyrosine, tryptophan, cystathionine and cystine were purchased from Phenomenex (Torrance, CA, USA).

### 2.3. Methods

#### 2.3.1. Palynological Analysis

The palynological analysis was conducted as follows: 2 g of bee pollen was prepared by dissolving and washing it in H_2_SO_4_ (5‰), and placed on the slide [33]. The slide was examined using a Primostar 3 KMAT Carl Zeiss microscope at 400× magnification. The pollen frequencies were determined based on the Louveaux et al. (1978) methodology, and were divided into four categories as: predominant pollen (>45% of the total pollen detected); secondary pollen (16–45%); minor important pollen (3–15%); minor pollen (<3%) [33].

#### 2.3.2. Determination of Routine Physicochemical Parameters: Moisture Content, Water Activity, pH and Free Acidity

- Moisture content

A total of 1 g of sample was weighed and heated at 103 °C for 2 h for start, weighted after cooling and heated again until constant weight was obtained [18].

- Water activity

Water activity was measured using a water activity meter AquaLab Lite (Decagon, USA).

- pH and free acidity

2 g of bee pollen was mixed with 5 mL of water, after homogenization the solution was filtered and titrated with NaOH 0.05 M [17]. The pH was determined on the same solution using a Metler Toledo pH meter Seven compact S210. All the measurements were carried out in triplicate.

#### 2.3.3. Proximate Composition of Bee Pollen

The nutritional value of the bee pollen involved the determination of total lipid (AOAC 920.85), total protein (AOAC 978.04) and ash (AOAC 920.85) [34]. All the measurements were carried out in triplicate. Carbohydrates were determined by difference. The energetic values of the bee pollen were determined as:

Energy (kcal) = 4 × (g protein + g carbohydrates) + 9 × (g lipid)(1)

#### 2.3.4. Determination of Organic Acids

A total of 0.5 g of bee pollen was mixed with 2.5 mL 4% metaphosphoric acid (*w*/*v*), after homogenization the solution was centrifuged for 10 min at 5000 rpm. The supernatant was filtered using a 0.45 µm filter prior analysis. The determination was carried out on a high performance liquid chromatograph Shimadzu (Kyoto, Japan) with diode array detector. The separation of the organic acids (gluconic acid, formic acid, butyric acid propionic acid, lactic acid and acetic acid, respectively) was made using a Phenomenex Kinetex^®^ 5 μm C18 100 Å HPLC Column 250 × 4.6 mm [35]. The mobile phase consisted of a mixture of 0.5% metaphosphoric acid and acetonitrile (50/50, *v*/*v*), and the flow rate was set at of 0.8 mL·min^−1^. The injection volume was set at 10 µL. The detector was set at 210 nm. The organic acids were expressed as mg/kg dry mater. All the measurements were carried out in triplicate.

#### 2.3.5. Determination of Free Sugars

A total of 1g of bee pollen was mixed with 20 mL of methanol and filled to 50 mL with water. The solution was centrifuged for 10 min at 5000 rpm; the supernatant was transferred into a 50 mL flask and filled with water. The solution was filtered using a 0.45 µm filter prior analysis. The separation of the free sugars (fructose, glucose, sucrose, melesitose and raffinose) was made on Schimadzu HPLC instrument (Kyoto, Japan) with RID (refractive index detector). A Phenomenex Luna^®^ Omega 3 μm SUGAR 100 Å HPLC Column 150 × 4.6 mm (Torrance, CA, USA) was used for the separation. The mobile phases were acetonitrile and water in a mixture of 80:20 (*v*/*v*). The flow rate was set at 1.3 mL·min^−1^; column and detector temperature was 30 °C and the sample volume injection was 10 µL. The free sugars were expressed as % reported to dry mater. All the measurements were carried out in triplicate.

#### 2.3.6. Bee Pollen Extracts for the Determination of Phenolic Compounds

The extraction procedure was carried out as follows: 0.1 g of bee pollen was mixed with 25 mL of 80% methanol and ultrasonicated for 30 min at 50 °C. After the heating, the solution was transferred into a centrifuge tube and centrifuged for 5 min at 5000 rpm. The supernatant was collected, filled up to 50 mL with 80% methanol and kept at 4 °C for TPC, TFC and individual phenolics compounds determination.

#### 2.3.7. Determination of Total Phenolic Content (TPC) and Total Flavonoid Content (TFC)

Total phenolic content (TPC) determination: 0.1 mL of bee pollen extract was mixed with 1.9 mL water, 0.1 mL of Folin Ciocalateau reagent, the mixture was homogenizated for 2 min and after it 0.8 mL of 5% sodium carbonate was added. The mixture was kept at 40 °C for 20 min and cooled down in an ice bath for stopping the reaction. The total phenolic content was determined at 750 nm, based on a gallic acid calibration curve expressing the results as gallic acid equivalent (mg GAE/g) dry mater [35]. All the measurements were carried out in triplicate.

Total flavonoid content (TFC) determination: 0.2 mL of bee pollen extract was mixed with 2 mL of methanol and 0.1 mL of 5% AlCl_3_ (prepared in methanol). The solution was left for 30 min at room temperature and its absorbance was measured at 425 nm. The concentration was determined based on a quercetin calibration curve expressing the results as mg quercetin equivalent/g (mg QE/g) dry mater [35]. All the measurement were carried out in triplicate.

#### 2.3.8. Determination of Individual Phenolic Compounds

The phenolic acids (rosmarinic acid, *p*-coumaric acid, chlorogenic acid, vanillic acid, caffeic acid, *p*-hydroxybenzoic acid, protocatechiuc acid, gallic acid) and flavonoids (kaempferol, quercetin, luteolin and myricetin) were determined from the methanolic extract using a high performance liquid chromatograph Shimadzu (Kyoto, Japan) with diode array detector. The separation was carried out on a Zorbax SP-C18 column, with 150 mm length, 4.6 mm i.d. 5 μm-diameter particle was used for the separation [35]. The separation of the compounds were realized on a system with 0.1% acetic acid (mobile phase A) and acetonitrile (mobile phase B) based on the elution range described by Palacios et al. [36] as: min 0—A 100%, min 6.66—B 5%, min 66.66—B 40% and min 74—B 80%. The flow rate was set at 1 mL·min^−1^, and the injection volume was 10 µL. Gallic acid, vanillic acid, protocatechuic acid and *p*-hydroxybenzoic acid were determined at 280 nm, and chlorogenic acid, *p*-coumaric acid, caffeic acid, rosmarinic acid, myricetin, quercetin, luteolin and kaempherol were determined at 320 nm, respectively. The phenolics compounds were expressed as mg/kg dry mater. All the measurements were carried out in triplicate.

#### 2.3.9. Determination of Total Free Amino Acids

For the extraction and identification of free amino acids, 1.75 ± 0.1 g of sample was mixed with 15 mL of 15% trichloroacetic acid (TCA). The pH of the mixture was adjusted to 2.2 (isoelectric precipitation point of the proteins) and the extract was further diluted to 25 mL with 15% trichloroacetic acid. Then, the supernatant was collected and filtered using 0.45 µm microfilters. A total of 100 µL of filtered supernatant was subjected to the determination of organic components, using the EZfaast GC-MS kit (Phenomenex, Torrance, CA, USA), following the protocol given by the manufacturer. Identification and separation of free amino acids was performed using a gas chromatograph coupled with a mass spectrometer (MS) equipped with a Zebron TM ZB-AAA column (10 m × 0.25 mm, film thickness: 0.25 μm). Injection: split 1:15, carrier gas: helium 1.1 mL/min, oven program: 30 °C/min from 110 °C to 320 °C. The MS parameters: source temperatrure 240 °C, scan range 45–450 *m*/*z*, sampling rate 3.5 scans/s. The identification of each amino acid was performed by calculating the area of each “peak” and comparing it with a standard consisting of 33 amino acids (alanine, sarcosine, glycine, α-aminobutyric acid, valine, β-amino isobutyric acid, internal standard, leucine, allo-isoleucine, isoleucine, threonine, serine, proline, asparagine, thioproline, aspartic acid, methionine, 3-hydroxyproline/4-hydroxyproline, phenylalanine, glutamic acid, α-aminoadipic acid, α-aminopimelic acid, glutamine, ornithine, glycyl-prolizine—2 isomers, proline-hydroxyproline, histidine, lysine, tyrosine, tryptophan, cystathionine and cystine). The results are expressed as µg/mg bee pollen; all the determinations were carried out in triplicate.

#### 2.3.10. Fatty Acids Determination Using GC-MS

Prior the analysis, the bee pollen was extracted for 48 h with n-hexane at room temperature [37]. The bee pollen oil (0.1 g) was mixed with 1 mL of n-hexane and 1 mL of 15% BF_3_ in methanol. The mixture was thermostated for 15 min at 60 °C in a water bath. The mixture was cooled to 20 °C and mixed with 5 mL of NaCl saturated solution, after mixing the solution was centrifuged for 5 min at 3000 rpm. The supernatant was filtered with a 0.45 µm nylon filter and kept at −20 °C prior analysis. The separation of the fatty acids methyl esters was carried out on SUPELCOWAX 10 column (60 m × 0.25 mm i.d., 0.25 μm film thickness; Supelco Inc., Bellefonte, PA, USA) using a Shimadzu GC-MS instrument (GC MS-QP 2010 Plus, Shimadzu, Japan) equipped with an AOC-01 auto-injector that was used to perform the gas chromatographic-mass spectrometric analyses. The initial oven temperature was 140 °C and was increased to 220 °C at a rate of 7 °C/min and then held at this temperature for 23 min. The flow rate of the carrier gas (He) and the split ratio were 0.8 mL/min and 1:24, respectively. Identification of FAMEs was done by comparing their retention times to those of known standards (37 component FAME Mix, Restek, Bellefonte, PA, USA, 35077) and the resulting mass spectra to the ones from our database (NIST MS Search 2.0) [17]. The results are expressed as µg/g bee pollen; all the determinations were carried out in triplicate.

#### 2.3.11. Determination of FT-IR

The bee pollen spectra in the wave number range of 4000–650 cm^−1^ was recorded using a Nicolet i-20 spectrophotometer (Thermo Scientific, Waltham, MA, USA) with ATR module in absorbance mode. The bee pollen spectra were analysed using Spectra Gryph–spectroscopy software (Version 1.2.11). Each sample was ground into powder and filtered with 200 mesh, pressed and analysed directly on the ATR module. Each spectra was the media of 32 scans at a resolution of 4 cm^−1^. All the measurements were carried out in triplicate.

### 2.4. Statistical Analysis

The results were submitted to analysis of variance (ANOVA) using Statgraphics Centurion XIX software (trial version, Statgraphics Technologies, Inc., The Plains, VA, USA). Tukey (HSD)/Analysis of the differences between the categories with a confidence interval of 95% was used. Principal component analysis (PCA) was performed using Unscrambler X software version 10.1 (Camo, Norway).

## 3. Results and Discussion

### 3.1. Botanical Origin of the Bee Pollen

The melissopalynological analysis of the bee pollen is presented in Table 1 and covers the identified plant families, species and genera presented. According to the analysis, neither one sample has reached 80% of a one single pollen to be considered as monofloral. From the 24 samples analyzed, 20 samples had a pollen which represented more than 45% of the pollen variability (five samples with *Helianthus annuus,* five samples with *Robinia pseudoacacia*, two samples with *Pinaceae* spp., two samples with *Quercus* spp., two samples with *Prunus* spp. and one sample with *Zea mays*, *Tillia* spp. *Crataegus monogyna Taraxacum* spp., respectively), while in the case of four samples neither one pollen type has reached the 45% level. Secondary pollens were found: *Robinia pseudoacacia*,* Tilia* spp. and *Helianthus annuus.* Minor pollens were observed *Fagus sylvatica*, *Corylus* spp., *Taraxacum* spp., *Vicia* spp., *Salicaceae* spp., *Sophora* spp., *Poaceae* spp., *Trifolium* spp., *Asteraceae* spp., *Urtisaceae* spp., *Pinaceae* spp., *Cucumber* spp., *Castaneae* spp., *Oleaceae* spp., *Allium* spp., *Plantago* spp., *Myrcia* spp., *Fireweed* spp., *Mimosa* spp., *Cucumber* spp. Pollen botanical origin from the pollen pellets may vary according to the region of collection, and vegetation available for bees at the collecting moment.

### 3.2. Routine Physicochemical Parameters: Moisture Content, Water Activity, pH and Free Aciditiy

In Table 2 are presented the physicochemical properties of bee pollen samples analyzed. The stability and the shelf life of bee pollen is influenced strongly by the pH and the titratable acidity; these two parameters are a good indicator of the dynamic microbial activity [14]. The bee pollen pH ranged between 3.90 and 5.84 with a mean of 4.60 (*p* < 0.05); the values are in agreement with those reported in the case of pollen from Tuscany, Portugal, Greece and India [8,27,38]. The free acidity ranged between 124.58 and 306.90 meq/kg with a mean of 215.51 meq/kg (*p* < 0.05); the magnitude of free acidity confirmed the acidic nature of the bee pollen. The free acidity of the samples were in agreement with those reported for Colombian bee pollen [39] and Brazilian bee pollen [40]. Moisture content ranged between 2.96% and 11.90% (*p* > 0.05), with an average of 5.00%, which confirms the dry characteristics labeled by the beekeepers on the products; the moisture content was much lower than those reported for bee pollen from Colombia, Italy and Spain [22], but in agreement with the levels determined in Brazilian bee pollen [40]. According to the literature, the moisture content of dry bee pollen should be between 6 to 8% to ensure the bee pollen quality and stability [29]. The water activity is correlated to the stability and shelf life of a product; a high water activity stimulates the growth of microorganisms (e.g., molds, yeasts) and can cause pollen toxicity due to mycotoxins formation [8]. The water activity ranged between 0.17 and 0.55 (*p* < 0.05), with an average of 0.29, in agreement with those reported for bee pollen from Portugal, Spain and Colombia [22,41].

### 3.3. Proximate Composition of Bee Pollen

Protein content ranged between 15.74 and 27.92% (*p* < 0.05) with an average of 22.31%, which confirms the important role of bee pollen for human nutrition. The level of proteins was in agreement with those reported from Brazil (12.28% to 27.07.%) [40], Spain (15.19–20.23%), Colombia (21.6%) and Italy (19.5%) [22]. Gardana et al. observed a lower content in terms of proteins (12.3%) for bee pollen from Spain [22]. The great variability of protein level in the 24 samples analyzed might be influenced by the floral sources, geographical origin and/or storage conditions.

Lipids are considered the third group of substances present in the bee pollen, after carbohydrates and proteins, and are vital for the generation of royal jelly. The lipids ranged between 2.24 and 7.30% (*p* < 0.05), with a mean concentration of 4.49%. The principal compounds are triglycerides, carotenoids and sterols [42].

Ash represents 2.29 to 4.02% (*p* < 0.05) of the bee pollen with a mean of 3.18%, in agreement with the literature [17]. In the case of energy value of the bee pollen it was 368.19 to 407.68 kcal/100 g (*p* < 0.05), with a mean of 390.66 kcal/100 g; this fact confirms the importance of this bee product for human nutrition.

### 3.4. Free Sugars of Bee Pollen

The carbohydrates represent the main compounds present in the bee pollen; the main compound determined was fructose followed by glucose, melesitose, trehalose, sucrose, maltose, raffinose and turanose in the bee pollen [4]. Fructose and glucose were in the same range as those reported for bee pollen from Colombia, Italy and Spain, while sucrose was much lower (5.1–6.2% for Colombia, Italy and Spain, and for the samples analysed in this study) [22]. In other studies, others sugars were reported such as arabinose, melibiose, isomaltose, melesitose, ribose, turanose and trehalose but they do not represent more than 1% of them [43]. The fructose/glucose ratio was between 1.13 and 2.43 (*p* < 0.05) with a mean ratio of 1.46. The Spanish bee pollen was reported to have a fructose/glucose ratio between 1.13 and 1.53 [44], the Polish bee pollen was reported to have a fructose/glucose ratio between 1.03 to 2.51 [30], while the Brazilian bee pollen was reported to have a fructose/glucose ratio between 1.01 to 2.24 [40]; one possible reason of the differences between the samples may attributed to the harvesting seasons [30,40].

### 3.5. Organic Acids of Bee Pollen

In this study the presence of gluconic acids, formic acid, lactic acid, acetic acid, succinic acid, propionic acid and butyric acid was investigated, and the results are presented in Table 3. As can be observed from the Table 3, the major organic acid was gluconic acid, followed by lactic acid, acetic acid and propionic acid. Formic acid, succinic acid and butyric acid were not present in any of the samples. Similar levels from gluconic acid, lactic acid and acetic acid were reported by Kalaycıoglu et al. [45] in the case of bee pollen from Turkey. The lactic acid presented in the bee pollen is probably the result of the fermentation process during the fermentation of carbohydrates using lactic acid bacteria present in the bees’ stomach [46]. The organic acids present preservation potential from foods and are promoted as a new generation instead of antibiotics, so the bee pollen can be considered a potential preservation agent [45,46]. The absence of formic acid and butyric acid represents a good indicator that the bee pollen is not contaminated with undesired microorganisms.

### 3.6. TPC, TFC and Individual Phenolics Compounds

In the Table 3 the total phenolic content, total flavonoid content and individual phenolics compounds of bee pollen are presented. The TPC ranged between 4.64 and 17.93 GAE mg/g (*p* < 0.05) while the TFC ranged between 4.93 and 20.45 QE mg/g. The significant differences (*p* < 0.05) were found between TPC and TFC of pollen extracts from different sources which might be due to variation in the botanical origins as well as different climatic conditions. The values of TPC were in the same range as those reported for Indian bee pollen (9.79–35.63 GAE mg/g) [10] and Brazilian pollen (6.50 to 29.20 mg GAE/g) [47]. Regarding the TFC, the values are in agreement with those reported for Brazilian bee pollen (0.30–17.50 mg QE/g) [47] and Indian bee pollen (9.72–15.62 GAE mg/g) [10]. From the phenolics compounds studied, gallic acid and caffeic acid were not reported in any samples studied. The protocatechiuc acid and *p*-hydroxybenzoic acid were only observed in one sample (S2—sample with more than 45% of the pollen from *Pinaceae* spp.), vanillic acid was reported in three samples (S1, S3 and S24) and chlorogenic acid in five samples (S1, S12, S18, S21 and S23). The other phenolics studied were more present in the pollen samples. The major compound found was quercetin (S19—sample with more than 45% of the pollen from *Prunus* spp.), followed by myricetin (S16—sample with more than 45% of the pollen from *Quercus* spp.) and kaempferol (S23—sample with more than 45% of the pollen from *Robinia pseudoacacia*). The Brazilian bee pollen [48] had a similar concentration of chlorogenic acid and vanillic acid; from 56 samples just two samples contained gallic acid and three caffeic acid, so their findings are similar to ours and we can conclude that bee pollen is not a source of this phenolic acids; the *p*-coumaric acid, quercetin and kaempferol were in a much lower concentration than those reported in this study which may be attributed to the different botanical or geographical origin of the samples. Thakur and Nanda [10] reported that flavonoids are influenced by the botanical origin of the bee pollen (catechin: 0.94–19.10 mg/100 g; rutin: 4.81–24.83 mg/100 g; quercetin: 3.14–15.94 mg/100 g; luteolin: 1.06–5.86 mg/100 g; kaempferol: 0.12–9.35 mg/100 g; and apigenin: 0.46–3.02 mg/100 g), quercetin and kaempferol were the major flavonoids reported by them but in lower concentration than in this study.

### 3.7. Total Free Amino Acid Composition

As the data presented in Table 4 states, 16 amino acids (eight essential amino acids and eight non-essential amino acids) were quantified in the bee pollen samples analyzed, and there was observed a significant difference in terms of amino acids concentration between the samples (*p* < 0.05). The total content of the amino acids determined for the bee pollen samples varied between 11.31 µg/mg (sample 21) and 45.99 µg/mg (sample 4). These values were comparable to those reported for the total free amino acid content of commercial bee pollen from Colombia (25.3 ± 1.0 mg/g), Italy (29.4 ± 0.7 mg/g) and Spain (30.8 ± 0.2 mg/g) [22], and higher than the total amino acids content of bee pollen from floral sources such as sunflower (12.20 g/100 g) and rape (12.25 g/100 g) [49]. From the data presented, in the samples analyzed in our study, the most abundant essential amino acids were histidine (values of 0.29–2.30 µg/mg), lysine (0.14–0.74 µg/mg) and phenylalanine (0.12–0.43 µg/mg). Histidine was found to predominate in the bee pollen collected during autumn, while high levels of lysine and phenylalanine were determined in bee pollen collected during winter [50]. The distribution of these essential amino acids in the analyzed bee pollen was therefore in accordance with the period when the samples were collected. Leucine, isoleucine and tryptophan were detected in low amounts, and similar findings were reported for monofloral bee pollen of *Geranium* botanical origin [15]. Glutamic acid was the main amino acid in all bee pollen samples, with values that varied between 0.34 and 18.77 µg/mg, followed by aspartic acid (0.01–14.08 µg/mg) and proline (2.79–7.19 µg/mg). Glutamic acid, aspartic acid and proline were also reported as the major amino acids in different bee pollen varieties (coconut, coriander, rapeseed and multifloral) from India [27]. The variation of the amino acid content was found to be influenced by both botanical origin and processing and storage conditions. In regard to the processing and storage conditions, previous studies reported that glutamic acid is the most abundant amino acid in bee pollen that is freshly collected, while proline is the free amino acid that is found in high amounts in well dried and stored bee pollen [22]. In the case of our study, the high content of glutamic acid was well correlated to the fact that the bee pollen samples analyzed in this study were freshly collected.

### 3.8. Fatty Acids Composition

For the bee pollen samples analyzed in this study, 19 fatty acids were quantified by the GC-MS method. The total content of fatty acids of the bee pollen samples varied between 81.69 µg/g (sample 10) and 645.72 µg/g (sample 24) (Table 4). The unsaturated fatty acids were predominant (UFA; 62.65–927.50 µg/g), of which levels of 7.51–88.69 µg/g were determined for monounsaturated fatty acids (MUFAs) and 43.86–838.82 µg/g for polyunsaturated fatty acids (PUFAs). The main MUFAs were oleic acid (C18:1 (Z)-octadec-9-enoic acid) and 11-eicosenoic acid (C20:1 (cis-11) (Z)-icos-11-enoic acid), which were also reported as prevalent in *Brassica napus* pollen from India [27]. Of the PUFAs, γ-linoleic acid (C18:3 (all-cis-6,9,12) octadeca-6,9,12-trienoic acid) and linoleic acid (C18:2 (all-cis-9,12) (9Z,12Z)-octadeca-9,12-dienoic acid) were determined in high levels in all pollen samples. By comparison, in 18 bee pollen samples from Turkey and Romania, Mărgăoan et al. [51] determined a higher content of α-linoleic acid than linoleic acid. In our study, α-linoleic acid was quantified only in samples 3 and 4. Palmitic acid (C16:0 hexadecanoic acid) and stearic acid (C18:0 octadecanoic acid) were determined as the main saturated fatty acids (SFA); these two fatty acids were also reported as the predominant saturated fatty acids in the bee pollen from 11 different floral sources from Taiwan [25] and in commercial bee pollen samples from Colombia, Italy and Spain [22]. C15:0 pentadecanoic acid and C17:0 heptadecanoic acid were determined in lower amounts and were found in less than half of the bee pollen samples analyzed in this study. When studying the fatty acids profile of bee pollen, the ratio between UFA and SFA is of great importance. It was considered that a value of the UFA/SFA ratio higher than 1 is characteristic of bee pollen with considerable nutritional value, while a value below 1 indicates degradation of unsaturated fatty acids due to storage and dehydration process [50]. For the 24 bee pollen samples analyzed in our study, the UFA/SFA ratio varied between 1.86 and 5.78 and was comparable with the values of 2.2–6.7 reported for the bee pollen from India [27] and the 1.9–2.2 UFA/SFA ratio calculated for commercial bee pollen from Colombia, Italy and Spain [22].

### 3.9. FTIR-ATR Spectroscopy of Pollen

**Figure 1:**
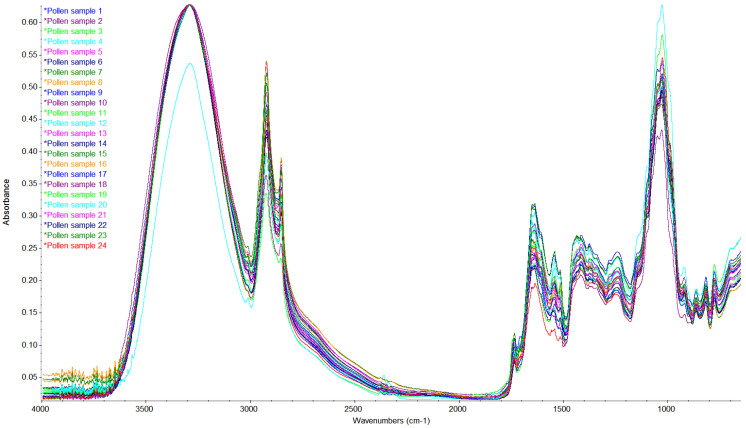
Bee pollen FT-IR spectra in the region 4000–650 cm^−1^.

The FTIR-ATR spectra of 24 pollen samples, recorded in absorbance mode in the mid-infrared region, is presented in Figure 1. The broad band around 3290 cm^–1^ that was observed in all pollen samples corresponded to O–H stretching vibration due to the presence of water [52,53]. As it was previously reported that the moisture content of fresh pollen varies between 21 and 30% [9], the presence of a broad band in this spectral region was expected. Between 3000 and 2850 cm^–1^, two peaks were identified in all the samples analyzed: a peak around 2920 cm^–1^ and one around 2850 cm^–1^, both assigned to C–H stretching, and mainly CH_2_ and CH_3_ vibrations of lipids, proteins and carbohydrates [52]. For one pollen sample (sample 4) a small peak was found at 2360 cm^–1^ and was also attributed to C–H stretching vibrations of lipids [54]. Furthermore, another signal corresponding to lipids, and namely the peak at 1740 cm^–1^, which is characteristic of stretching vibrations of C=O groups, was prominent in some pollen samples; previous research found that this signal shows large variation within pollen samples of related plant species [55]. In the spectra of all pollen samples, peaks were observed at 1650 cm^–1^ and 1540 cm^–1^ that were attributed to stretching vibrations of amide I and II [56]. Characteristic of all samples was also the peak at 1414 cm^–1^ that was assigned to asymmetric in-plane bending of the –CH_3_ group [53]. The peak in the region between 1350 and 1200 cm^–1^ was assigned to amide III, and more precisely an in-phase combination of N–H deformation vibrations and C–N stretching vibrations [56]. All samples had high absorption peaks around 1030 cm^–1^ that corresponded to stretching vibrations of saccharides and proteins, and were also reported for crude pollen and defatted pollen samples in our previous study on the extraction of polyphenols from crude pollen [17]. In the spectral range between 1200 and 500 cm^–1^, which is considered the fingerprint region of pollen, the peaks observed for the analyzed samples were due to C–O and C–C stretching vibrations, and their variation among pollen samples indicated differences in the saccharide, protein and lipid composition. This overlapped the region of 1500–800 cm^–1^, where characteristic signals were attributed to C–O and C–C stretching vibrations of flavonoids and phenolic compounds [57].

### 3.10. Principal Component Analysis

**Figure 2:**
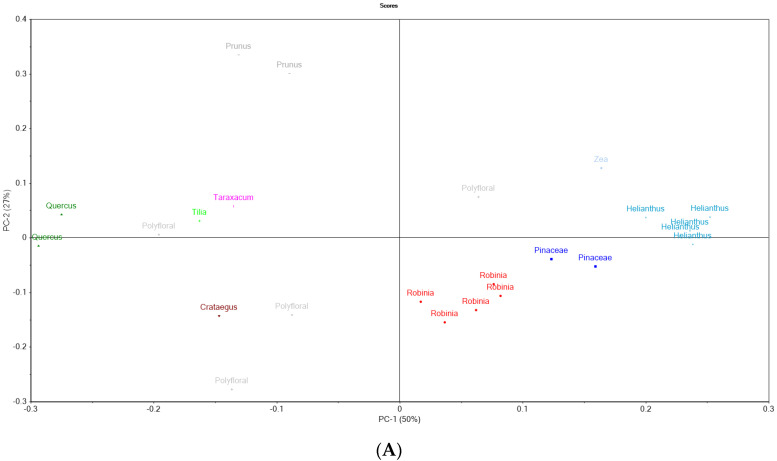

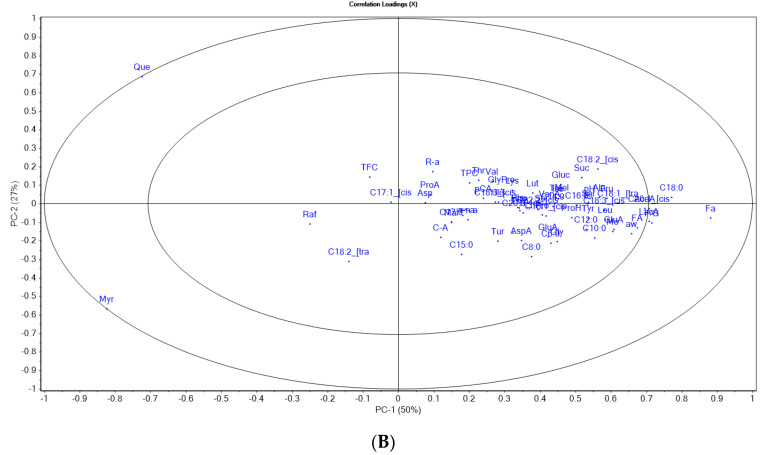
Principal component analysis scores (**A**) and loadings (**B**) of the bee pollen based on their physicochemical, organic acids, sugars, TPC, TFC, phenolics compounds, amino acids, fatty acids: a—scores, b—loadings, Fa-free acidity, Mo—moisture content, Fa-lipids, p-protein content, Fru-fructose, Glu-glucose, Suc-sucrose, Tur-turanose, Mal-maltose, Tre-trehalose, Mel-melesitose, Raf-raffinose, GluA-gluconic acid, LacA-lactic acid, AceA-acetic acid, ProA- propionic acid, TPC-total phenolic content, TFC-total flavone content, p-a—protocatecuic acid, 4-h-a—p-hydroxibenzoic acid, C-a—chlorogenic acid, p-c-A—p-coumaric acid, R-a—rosmarinic acid, V-A vanillic acid, Ala-alanine, Sar-Sarcosine, Gly-glicine, Val-valine, Leu-leucine, Iso—isoleucine, Thr-threonine, Ser-Serine, Pro-Proline, Asp-asparagine, AspA-aspartic acid, Phe-phenylalanine, GluA-glumatic acid, His-histidine, Lys-lysine, Tyr-tyrosine, Tryp-tryptophan.

The PCA was conducted based on the analysis discussed in order to discriminate the bee pollen samples based on their botanical origin. The first two principal components (PC1 and PC2) explained 77% of the data variance (PC1 explained 50% of the data variance, while PC2 explained 27% of the data variance). The PC1 is influenced strongly by lipids and negatively by raffinose and C18:2, the PC2 is influenced positively by quercetin while myricetin influences it negatively. Propionic acid, C17:1, TFC and asparagine do not influence the projection of the scores due to their closeness to the origins of the two PC. As can be seen in Figure 2A, the samples with high percentage of *Helianthus annus*, *Robinia pseudoacacia*, *Pinaceae* spp., *Quercus* spp. and *Prunus* spp. formed clusters which confirms that the analysis carried out is useful for their discrimination. Regarding the polyfloral bee pollen it can be observed that the samples are near on the other one but they include in their region the bee pollen from *Crataegus monogyna*, *Tilia* spp. and *Taraxacum*. The *Quercus* spp. and *Crataegus monogyna* exhibited a high myricetin content, while *Prunus* spp. exhibited a high quercetin content. The *Helianthus annus* samples exhibited a high free acidity, C18:0, F/G, lactic acid, lipids and C20:1. The polyfloral samples exhibited a high by raffinose and C18:2, myricetin and raffinose (Figure 2B). The *Robinia pseudoacacia* pollen samples were associated with C15:0, chlorogenic acid, turanose, maltose, C17:0, and *p*-hydroxibenzoic acid. A high positive correlation between moisture content and water activity (r = 0.898), moisture content and F/G (r = 0.818) was observed. The lipids content was positively correlated with C18:1 [trans-9]) + (C18:1 [cis-9] (r = 0.812), C18:2 [cis-9,12] (r = 0.898), C18:3 [cis-6,9,12] (r = 0.852), C22:2 [cis-13,16] (r = 0.772), MUFA (r = 0.796), PUFA (r = 0.870), UFA (r = 0.869) and SFA (r = 0.825). The TPC and TFC were correlated positively with quercetin (r = 0.579, r = 0.705) and kaempferol (r = 0.705, r = 0.679). The protein content was correlated with proline (r = 0.309) and asparagine (r = 0.302). The energetic value was correlated negatively with moisture content (r = −0.527), and positively with lipids (r = 0.587), sucrose (r = 0.328), MUFA (r = 0.620) and PUFA (r = 0.591).

## 4. Conclusions

In this study, we established that bee pollen is a rich source of protein, polyphenols, fatty acids, organic acids and amino acids. The organic acids (gluconic, lactic, acetic and propionic acids) provide antimicrobial properties for foods and are promoted as a new generation alternative to antibiotics, so bee pollen can be considered a potential preservation agent. The high protein content was observed in *Robinia pseudoacacia*, the high content of lipids was observed in *Robinia pseudoacacia* pollen, the high fructose content in *Prunus* spp. pollen while the high F/G ratio was observed in *Pinaceae* spp. pollen. The high TPC was observed in *Prunus* spp. pollen, the high TFC was observed in *Robinia pseudoacacia* pollen, the high free amino acid content was observed in *Pinaceae* spp. pollen, and the high content of PUFA was reported in *Taraxacum* spp. pollen. A total of 16 amino acids (eight essential amino acids and eight non-essential amino acids) were quantified in the bee pollen samples analyzed. Predominant were the unsaturated fatty acids (UFA; 62.65–927.50 µg/g), of which levels of 7.51–88.69 µg/g were determined for monounsaturated fatty acids (MUFAs) and 43.86–838.82 µg/g for polyunsaturated fatty acids (PUFAs). According to the data obtained, bee pollen can be considered a complex matrix with a high potential as food supplement or source of bioactive compounds for the pharmaceutical industry.

## Figures and Tables

**Table 1:** Palynological analysis: Plant species giving the predominant, secondary, important minor and minor pollen in the analyzed bee samples.

Sample Code	Dominant Pollen (>45%)	Secondary Pollen (16–44%)	Important Minor Pollen (4–15%)	Minor Pollen (<3%)
S1	*Helianthus annuus*	*-*	*Robinia pseudoacacia* *Zea mays* *Tilia* spp. *Fagus sylvatica*	*Quercus* spp. *Betulus pendula*
S2	*Pinaceae* spp.	*-*	*Corylus* spp. *Taraxacum* spp. *Vicia* spp. *Helianthus annuus*	*Carduus* spp. *Ambrosia* spp.
S3	*Helianthus annuus*	*-*	*Robinia pseudoacacia* *Tilia* spp.	*Conicum* spp. *Vicia* spp. *Pruunus spinosa*
S4	*Tillia* spp.		*Allium* spp. *Helianthus annuus* *Asteraceae* spp.	*Brassicaceae* spp. *Fagopyrum* spp.
S5	*-*	*-*	*Helianthus annuus* *Taraxacum* spp. *Quercus* spp. *Zea mays*	*Prunus* spp.
S6	*-*	*-*	*Trifolium* spp. *Robinia pseudoacacia* Helianthus annuus *Poaceae* spp. *Vicia* spp.	*Fabaceae* spp. *Conicum* spp. *Fagus* spp. *Ulmu* spp.
S7	*Robinia pseudoacacia*	*-*	*Asteraceae* spp. *Urtisaceae* spp.	*Rosaceae* spp. *Prunus* spp.
S8	*Quercus* spp.	*-*	*Pinaceae* spp.	*Castaneae* spp.
S9	*Robinia pseudoacacia*	*-*	*Cucumber* spp. *Castaneae* spp. *Oleaceae* spp.	*Rosaceae* spp. *Prunus* spp.
S10	*-*	*-*	*Trifolium* spp. *Robinia pseudoacacia* *Urticaceae* spp. *Castanea* spp.	*Quercus* spp.
S11	*Zea mays*		*Trifolium* spp.	*Quercus* spp.
S12	*Helianthus annuus*	*Robinia pseudoacacia*	*-*	*Vicia* spp. *Pruunus spinosa* *Tilia* spp.
S13	*Crataegus monogyna*	*-*	*Helianthus annuus* *Taraxacum* spp *Quercus* spp.	*Robinia pseudoacacia*
S14	*Helianthus annuus*	*Tilia* spp.	*Asteraceae* spp.	*Taraxacum* spp.
S15	*Taraxacum* spp.	*-*	*Plantago* spp. *Quercus* spp.	
S16	*Quercus* spp.	*-*	*Taraxacum* spp. *Castaneae* spp.	*Ambrosia* spp. *Helianthus annuus*
S17	*Robinia pseudoacacia*	*Helianthus annuus*	*Taraxacum* spp. *Castaneae* spp.	*Tilia* spp.
S18	*Robinia pseudoacacia*	*Helianthus annuus*	*Tilia* spp.	*Taraxacum* spp. *Castaneae* spp.
S19	*Prunus* spp.	*-*	*Taraxacum* spp. *Quercus* spp.	*Robinia pseudoacacia* *Helianthus annuus* *Teucrium* spp.
S20	*Pinaceae* spp.	*-*	*Fagaceae* spp.	*Prunus* spp. *Asteraceae* spp. *Gramineae* spp.
S21	*-*	*-*	*Zea mays* *Myrcia* spp. *Helianthus annuus* *Fireweed* spp. *Mimosa* spp.	*Salix* spp. *Taraxacum* spp.
S22	*Prunus* spp.		*Cucumber* spp.	*Quercus* spp.
S23	*Robinia pseudoacacia*	*-*	*Salicaceae* spp. *Sophora* spp. *Poaceae* spp.	*Humulus* spp. *Salicaceae* spp. *Allium* spp.
S24	*Helianthus annuus*	*-*	*Robinia pseudoacacia* *Tilia* spp.	*Quercus* spp. *Vicia* spp. *Pruunus spinosa*

**Table 2:** Physicochemical parameters (moisture content, water activity, pH and free acidity), proximate composition and free sugars of bee pollen samples (mean values and standard deviation in brackets).

Parameter	*Crataegus monogyna*	*Helianthus annuus*	*Pinaceae* spp.	Polyfloral	*Prunus* spp.	*Quercus* spp.	*Robinia pseudoacacia*	*Taraxacum* spp.	*Tillia* spp.	*Zea mays*	F-Value
pH	4.57 (0.07) ^a,b,c^	4.36 (0.27) ^a,b^	4.12 (0.30) ^a^	4.85 (0.23) ^a,b,c^	5.19 (0.69) ^c^	4.69 (0.14) ^a,b,c^	4.24 (0.23) ^a,b^	4.96 (0.07) ^b,c^	4.85 (0.07) ^a,b,c^	4.53 (0.06) ^a,b,c^	6.4 ***
Free acidity (meq/kg)	233.4 (3.3) ^a,b^	187.7 (33.0) ^a^	272.79 (26.90) ^b^	179.6 (16.5) ^a^	199.3 (87.8) ^a,b^	252.0 (59.8) ^b^	249.13 (24.41) ^b^	214.2 (3.0) ^a,b^	194.0 (2.7) ^a,b^	127.5 (1.8) ^a^	5.2 ***
Moisture content (%)	4.92 (0.07) ^a,b^	5.07 (1.62) ^a,b^	8.84 (3.40) ^b^	4.74 (1.12) ^a^	4.86 (2.05) ^a,b^	4.89 (1.16) ^a,b^	4.34 (0.57) ^a^	3.32 (0.05) ^a^	4.50 (0.06) ^a^	2.90 (0.04) ^a^	3.8 **
a_w_	0.27 (0.01) ^a^	0.30 (0.10) ^a,b^	0.48 (0.08) ^b^	0.27 (0.06) ^a^	0.24 (0.08) ^a^	0.29 (0.09) ^a,b^	0.28 (0.05) ^a^	0.17 (0.01) ^a^	0.28 (0.01) ^a^	0.20 (0.01) ^a^	4.2 ***
Protein. d.m. (%)	22.66 (0.32) ^b,c,d^	18.03 (1.05) ^a,b^	23.09 (2.48) ^c,d^	23.06 (1.35) ^c,d^	24.54 (1.40) ^c,d^	26.86 (0.95) ^d^	23.13 (2.82) ^c,d^	23.71 (0.34) ^c,d^	21.14 (0.30) ^b,c^	15.58 (0.22) ^a^	13.7 ***
Lipids d.m. (%)	2.62 (0.04) ^a,b^	3.72 (1.11) ^a,b^	6.05 (1.36) ^c^	4.31 (1.01) ^a,b^	5.25 (0.43) ^b,c^	3.28 (0.87) ^a,b^	6.02 (0.89) ^c^	3.51 (0.05) ^a,b^	3.30 (0.05) ^a,b^	2.22 (0.03) ^a^	8.7 ***
Ash (%)	3.23 (0.05) ^b,c^	2.57 (0.14) ^a^	3.16 (0.45) ^b^	3.30 (0.23) ^b,c^	3.50 (0.13) ^b,c^	3.83 (0.18) ^e^	3.32 (0.40) ^b,c^	3.44 (0.05) ^b,c^	3.03 (0.04) ^a,b^	2.27 (0.03) ^a^	12.4 ***
Energy kcal/100 g	377.0 (5.3) ^a^	384.8 (8.0) ^a^	380.30 (18.77) ^a^	386.2 (5.6) ^a^	389.9 (7.5) ^a,b^	378.1 (4.4) ^a^	396.49 (7.49) ^b^	387.0 (5.5) ^a,b^	382.9 (5.4) ^a^	386.6 (5.3) ^a,b^	2.6 *
Fructose d.m. (%)	18.82 (0.27) ^a,b^	19.49 (1.54) ^a,b,c^	20.00 (1.11) ^a,b,c^	19.59 (0.64) ^a,b,c^	21.31 (3.03) ^c^	20.08 (0.25) ^a,b,c^	18.46 (0.98) ^a^	19.44 (0.28) ^a,b,c^	21.44 (0.31) ^c^	20.68 (0.30) ^b,c^	2.4 *
Glucose d.m. (%)	12.78 (0.18) ^a,b^	14.50 (2.75) ^a,b^	9.49 (1.03) ^a^	14.91 (1.78) ^a,b^	16.48 (5.47) ^b^	14.52 (0.21) ^a,b^	11.53 (1.25) ^a,b^	17.19 (0.25) ^b^	16.19 (0.23) ^b^	17.40 (0.25) ^b^	4.9 ***
Sucrose d.m. (%)	0.13 (0.01) ^a^	0.48 (0.33) ^a^	0.73 (0.11) ^a,b^	0.79 (0.22) ^a,b^	0.43 (0.29) ^a^	0.34 (0.39) ^a^	1.23 (0.43) ^b^	0.23 (0.01) ^a^	1.45 (0.02) ^b^	0.84 (0.01) ^a,b^	7.2 ***
Turanose d.m. (%)	0 (0) ^a^	0.10 (0.13) ^a^	0 (0) ^a^	0.08 (0.14) ^a^	0 (0) ^a^	0.07 (0.09) ^a^	0.05 (0.01)	0 (0) ^a^	0(0) ^a^	0(0) ^a^	0.6 ns
Maltose d.m. (%)	0.21 (0.01) ^a^	0.30 (0.32) ^a^	0.77 (0.07)	0.61 (0.14) ^a^	0.45 (0.09) ^a^	1.06 (0.97) ^a^	0.58 (0.25)	0.46 (0.01) ^a^	0.92 (0.01) ^a^	0.50 (0.01) ^a^	1.3 ns
Trehalose d.m. (%)	0.37 (0.01) ^a^	1.14 (0.81) ^a^	1.21 (0.36)	1.13 (0.04) ^a^	1.93 (1.27) ^a^	1.16 (0.60) ^a^	0.85 (0.23)	0.96 (0.01) ^a^	1.03 (0.01) ^a^	1.03 (0.01) ^a^	1.5 ns
Melesitose d.m. (%)	2.99 (0.04) ^a^	2.13 (0.31) ^a^	0.90 (0.84)	1.21 (1.30) ^a^	2.41 (0.73) ^a^	1.86 (2.14) ^a^	1.56 (0.89)	2.29 (0.03) ^a^	2.60 (0.04) ^a^	1.53 (0.02) ^a^	1.7 ns
Raffinose d.m. (%)	0.16 (0.01) ^a,b^	0.09 (0.09) ^a^	0.29 (0.19) ^a,b,c,d^	0.22 (0.14) ^a,b,c^	0.20 (0.20) ^a,b,c^	0.56 (0.57) ^d^	0.07 (0.06) ^a^	0.44 (0.01) ^b,c,d^	0.50 (0.01) ^c,d^	0.06 (0.01) ^a^	3.4 **
F/G	1.46 (0.02) ^b,c^	1.37 (0.23) ^a,b,c^	2.11 (0.34) ^d^	1.31 (0.11) ^a,b,c^	1.35 (0.26) ^a,b,c^	1.37 (0.02) ^a,b,c^	1.60 (0.15) ^c^	1.12 (0.02) ^a^	1.31 (0.02) ^a,b,c^	1.18 (0.02) ^a,b^	8.4 ***

d.m.—dry matter. ^a–e^ different letters in the same column indicate differences between samples (*p* < 0.05). ns-not significant (*p* > 0.05), *—*p* < 0.05, **—*p* < 0.01, ***—*p* < 0.001.

**Table 3:** Organic acids, total phenolic content, total flavonoid content and individual phenolics compounds of bee pollen (mean values and standard deviation in brackets).

	*Crataegus monogyna*	*Helianthus annuus*	*Pinaceae* spp.	Polyfloral	*Prunus* spp.	*Quercus* spp.	*Robinia pseudoacacia*	*Taraxacum* spp.	*Tillia* spp.	*Zea mays*	F-Value
Gluconic acid (g/kg)	33.78 (0.48) ^a,b,c^	21.68 (1.05) ^a,b^	34.47 (0.60) ^b,c^	30.00 (5.04) ^a,b,c^	29.37 (13.89) ^a,b,c^	22.22 (4.48) ^a,b^	36.33 (7.98) ^c^	24.88 (0.35) ^a,b,c^	26.24 (0.37) ^a,b,c^	14.03 (0.20) ^a^	4.9 ***
Lactic acid (g/kg)	0.67 (0.01) ^a,b^	0.61 (0.16) ^a^	1.10 (0.01) ^b^	0.69 (0.13) ^a,b^	0.74 (0.41) ^a,b^	0.67 (0.10) ^a,b^	0.76 (0.13) ^a,b^	0.54 (0.01) ^a^	0.77 (0.01) ^a,b^	0.47 (0.01) ^a^	3.8 **
Acetic acid (g/kg)	0.26 (0.01) ^a^	0.61 (0.23) ^a,b^	1.20 (0.01) ^c^	0.44 (0.09) ^a,b^	0.71 (0.52) ^b^	0.28 (0.14) ^a,b^	0.49 (0.41) ^a,b^	0.28 (0.01) ^a,b^	0.58 (0.01) ^a,b^	0.28 (0.01) ^a,b^	10.4 ***
Propionic acid (g/kg)	0.43 (0.01) ^a^	0.11 (0.23) ^a^	0 (0) ^a^	0.05 (0.09) ^a^	0.45 (0.52) ^a^	0.29 (0.14) ^a^	0.37 (0.31) ^a^	0.04 (0.01) ^a^	0.26 (0.01) ^a^	0.13 (0.01) ^a^	1.6 ns
TPC (GAE mg/g)	8.73 (0.12) ^a,b,c^	7.56 (3.02) ^a,b^	12.39 (0.17) ^a,b,c,d^	13.53 (2.16) ^b,c,d^	15.74 (2.33) ^d^	15.52 (1.17) ^d^	14.11 (3.08) ^c,d^	16.45 (0.24) ^d^	14.83 (0.21) ^c,d^	7.10 (0.10) ^a^	9.3 ***
TFC (QE mg/g)	8.55 (0.12) ^a,b,c^	5.95 (1.04) ^a^	11.03 (0.16) ^b,c,d^	13.97 (1.57) ^d,e,f^	17.37 (3.33) ^f^	15.68 (0.42) ^e,f^	18.81 (2.19) ^c,d,e^	16.39 (0.23) ^e,f^	14.79 (0.21) ^d,e,f^	6.28 (0.09) ^a,b^	29.1 ***
P-A	0 (0) ^a^	0 (0) ^a^	88.93 (0) ^a^	0 (0) ^a^	0 (0) ^a^	0 (0) ^a^	0 (0) ^a^	0 (0) ^a^	0 (0) ^a^	0 (0) ^a^	3.8 ***
p-H-A	0 (0) ^b^	0 (0) ^b^	21.02 (0) ^a^	0 (0) ^b^	0 (0) ^b^	0 (0) ^b^	0 (0) ^b^	0 (0) ^b^	0 (0) ^b^	0 (0) ^b^	3.9 ***
V-A (mg/kg)	0 (0) ^a^	21.19 (1.20) ^b^	0 (0) ^a^	0 (0) ^a^	0 (0) ^a^	0 (0) ^a^	0 (0) ^a^	0 (0) ^a^	0 (0) ^a^	0 (0) ^a^	4.1 ***
C-A	0 (0) ^a^	0.78 (0.10) ^a^	0 (0) ^a^	3.82 (1.08) ^a^	0 (0) ^a^	0 (0) ^a^	3.63 (4.90) ^a^	0 (0) ^a^	0 (0) ^a^	0 (0) ^a^	0.8 ns
*p*-C-A (mg/kg)	2.92 (0.04) ^a^	24.75 (17.36) ^a^	139.79 (3.82) ^a,b^	79.26 (66.99) ^a^	92.74 (49.07) ^a^	27.78 (0.33) ^a^	238.97 (67.93) ^b^	23.36 (0.33) ^a^	168.70 (2.41) ^a,b^	18.58 (0.27) ^a^	9.5 ***
R-A	0 (0) ^a^	2.01 (1.25) ^a,b^	0 (0) ^a^	11.65 (12.61) ^a,b^	3.63 (4.19) ^a,b^	26.08 (14.77) ^b^	14.92 (12.89) ^a,b^	14.50 (0.21) ^a,b^	22.75 (0.32) ^a,b^	85.14 (1.22) ^c^	16.8 ***
Myricetin (mg/kg)	397.49 (5.68) ^b,c,d,e^	33.36 (21.85) ^a,b^	209.11 (4.54) ^a,b,c,d^	558.08 (160.75) ^d,e^	183.92 (22.48) ^a,b,c^	712.13 (211.67) ^e^	256.93.16 (206.45) ^a,b,c,d^	284.01 (4.06) ^a,b,c,d^	439.01 (6.27) ^c,d,e^	0 (0) ^a^	12.7 ***
Luteolin (mg/kg)	0 (0) ^a^	13.45 (10.73) ^a,b,c^	10.79 (0.31) ^a,b^	9.75 (8.05) ^a,b^	3.08 (3.56) ^a^	0 (0) ^a^	26.22 (17.50) ^b,c^	0 (0) ^a^	33.46 (0.48) ^c^	0 (0) ^a^	3.1 **
Quercitin (mg/kg)	126.38 (1.81) ^a^	22.11 (40.39) ^a^	28.14 (0.80) ^a^	172.43 (125.23) ^a^	757.22 (454.34) ^c^	686.07 (35.27) ^b,c^	105.063 (100.80) ^a^	296.34 (4.23) ^a,b^	381.56 (5.45) ^a,b,c^	71.36 (1.02) ^a^	14.2 ***
Kaempferol (mg/kg)	126.45 (1.81) ^a,b^	52.22 (26.18) ^a^	186.77 (2.45) ^a,b^	269.20 (148.03) ^a,b^	283.42 (56.03) ^a,b^	269.41 (4.81) ^a,b^	363.19 (181.96) ^b,c^	652.38 (9.32) ^c^	406.47 (5.81) ^b,c^	323.18 (4.62) ^a,b^	8.6 ***

TPC—total phenolic content, TFC—total flavone content, p-a—protocatechuic acid, p-h-a—p-hydroxybenzoic acid, C-a—chlorogenic acid, p-c-A—p-coumaric acid, R-a—rosmarinic acid, V-A vanillic acid. ^a–f^ different letters in the same column indicate differences between samples (*p* < 0.05). ns-not significant (*p* > 0.05), **—*p* < 0.01, ***—*p* < 0.001.

**Table 4:** Amino acids profile and fatty acids composition of bee pollen (mean values and standard deviation in brackets).

	*Crataegus monogyna*	*Helianthus annuus*	*Pinaceae* spp.	Polyfloral	*Prunus* spp.	*Quercus* spp.	*Robinia pseudoacacia*	*Taraxacum* spp.	*Tillia* spp.	*Zea mays*	F-Value
Amino acids, μg/mg bee pollen											
Valine	0.18 (0.01) ^a^	0.14 (0.07) ^a^	0.12 (0.02) ^a^	0.19 (0.08) ^a^	0.22 (0.03) ^a^	0.24 (0.09) ^a,b^	0.20 (0.03) ^a^	0.41 (0.01) ^b^	0.21 (0.01) ^a^	0.18 (0.01) ^a^	5.1 ***
Leucine	0.08 (0.01) ^a,b^	0.08 (0.01) ^a,b^	0.08 (0.03) ^a,b^	0.09 (0.01) ^a,b^	0.06 (0.03) ^a^	0.08 (0.01) ^a,b^	0.11 (0.02) ^b^	0.09 (0.01) ^a,b^	0.09 (0.01) ^a,b^	0.09 (0.01) ^a,b^	3.5 **
Isoleucine	0.12 (0.01) ^a^	0.16 (0.06) ^a^	0.13 (0.03) ^a^	0.17 (0.04) ^a^	0.16 (0.05) ^a^	0.20 (0.04) ^a^	0.16 (0.03) ^a^	0.24 (0.01) ^a^	0.21 (0.01) ^a^	0.16 (0.01) ^a^	1.8 ns
Threonine	0 (0) ^a^	0.10 (0.03) ^b^	0.12 (0.04) ^b,c^	0.21 (0.05) ^d,e^	0.16 (0.06) ^b,c,d^	0.20 (0.03) ^c,d,e^	0.15 (0.02) ^b,c,d^	0.27 (0.01) ^e^	0.09 (0.01) ^b^	0.22 (0.01) ^d,e^	15.2 ***
Phenylalanine	0.25 (0.01) ^a,b^	0.23 (0.08) ^a,b^	0.18 (0.05) ^a^	0.29 (0.09) ^a,b^	0.21 (0.08) ^a^	0.28 (0.02) ^a,b^	0.19 (0.04) ^a^	0.40 (0.01) ^b^	0.30 (0.01) ^a,b^	0.21 (0.01) ^a^	3.2 **
Histidine	1.18 (0.02) ^a,b,c^	1.28 (0.71) ^a,b,c^	0.45 (0.18) ^a^	0.97 (0.13) ^a,b,c^	0.84 (0.44) ^a,b,c^	1.83 (0.52) ^c^	0.62 (0.26) ^a,b^	1.64 (0.02) ^b,c^	1.09 (0.02) ^a,b,c^	0.50 (0.01) ^a^	4.8 ***
Lysine	0.40 (0.01) ^a,b,c^	0.35 (0.23) ^a,b,c^	0.01 (0.00) ^a^	0.32 (0.20) ^a,b,c^	0.10 (0.11) ^a,b^	0.47 (0.20) ^b,c^	0.29 (0.23) ^a,b,c^	0.25 (0.01) ^a,b,c^	0.73 (0.01) ^c^	0.46 (0.01) ^a,b,c^	3.3 **
Tryptophan	0.18 (0.01) ^a^	0.12 (0.07) ^a^	0.11 (0.01) ^a^	0.18 (0.02) ^a^	0.14 (0.01) ^a^	0.16 (0.01) ^a^	0.16 (0.04) ^a^	0.18 (0.01) ^a^	0.17 (0.01) ^a^	0.16 (0.01) ^a^	1.5 ns
Alanine	0.31 (0.01) ^a,b^	0.26 (0.07) ^a,b^	0.21 (0.03) ^a^	0.26 (0.04) ^a,b^	0.22 (0.01) ^a^	0.31 (0.04) ^a,b^	0.38 (0.07) ^b^	0.26 (0.01) ^a,b^	0.31 (0.01) ^ab^	0.32 (0.01) ^a,b^	5.6 ***
Sarcosine	0.04 (0.01) ^a^	0.03 (0.01) ^a^	0.03 (0.00) ^a^	0.03 (0.01) ^a^	0.03 (0.01) ^a^	0.03 (0.01) ^a^	0.03 (0.01) ^a^	0.04 (0.01) ^a^	0.04 (0.01) ^a^	0.04 (0.01) ^a^	1.6 ns
Glycine	0.08 (0.01) ^a^	0.05 (0.03) ^a^	0.06 (0.03) ^a^	0.07 (0.02) ^a^	0.03 (0.01) ^a^	0.06 (0.02) ^a^	0.06 (0.02) ^a^	0.04 (0.01) ^a^	0.04 (0.01) ^a^	0.04 (0.01) ^a^	1.9 ns
Serine	1.32 (0.02) ^d^	0.71 (0.29) ^a,b,c^	0.33 (0.11) ^a^	0.76 (0.16) ^a,b,c^	0.49 (0.28) ^a,b^	0.85 (0.10) ^a,b,c,d^	0.85 (0.22) ^a,b,c^	0.67 (0.01) ^a,b,c^	0.97 (0.01) ^b,c,d^	1.12 (0.02) ^c,d^	5.4 ***
Proline	7.12 (0.10) ^c^	3.66 (0.46) ^a,b^	3.32 (0.64) ^a,b^	3.74 (0.37) ^a,b^	3.78 (0.86) ^a,b^	4.92 (0.27) ^b^	4.92 (0.94) ^a,b^	4.88 (0.07) ^b^	4.25 (0.06) ^a,b^	3.20 (0.05) ^a^	8.7 ***
Asparagine	1.34 (0.02) ^a,b,c^	0.74 (0.53) ^a,b^	0.50 (0.01) ^a^	1.22 (0.50) ^a,b,c^	1.03 (0.29) ^a,b,c^	1.64 (0.03) ^b,c^	1.64 (0.18) ^a^	1.71 (0.01) ^c^	0.99 (0.01) ^a,b,c^	0.77 (0.01) ^a,b^	5.9 ***
Aspartic acid	6.94 (0.10) ^a,b,c^	5.62 (4.92) ^a,b,c^	10.77 (0.86) ^b,c^	3.32 (3.55) ^a,b,c^	1.02 (0.89) ^a^	2.57 (2.51) ^a,b^	2.57 (2.99) ^a,b,c^	3.08 (0.04) ^a,b,c^	11.64 (0.17) ^c^	0.38 (0.01) ^a^	4.2 **
Glutamic acid	11.50 (0.16) ^a,b,c^	8.35 (6.17) ^a,b,c^	14.82 (1.49) ^b,c^	5.12 (5.08) ^a,b^	1.31 (0.96) ^a^	4.20 (2.16) ^a,b^	4.20 (4.01) ^a,b^	3.06 (0.04) ^a^	17.83 (0.25) ^c^	3.88 (0.06) ^a,b^	4.9 ***
Tyrosine	0.25 (0.01) ^a,b^	0.21 (0.05) ^a,b^	0.18 (0.01) ^a^	0.21 (0.04) ^a,b^	0.15 (0.01) ^a^	0.20 (0.05) ^a,b^	0.15 (0.03) ^a,b^	0.18 (0.01) ^a,b^	0.22 (0.01) ^b^	0.16 (0.01) ^a,b^	2.5 *
Total AA content	31.26 (0.45) ^b,c^	22.11 (9.49) ^a,b,c^	31.40 (2.24) ^b,c^	17.13 (8.14) ^a,b^	9.94 (1.28) ^a^	18.23 (4.67) ^a,b^	8.84 (7.22) ^a,b^	17.38 (0.25) ^a,b^	28.41 (0.56) ^c^	11.87 (0.17) ^a^	5.4 ***
Fatty acids, μg/g bee pollen											
(C6:0)	0.04 (0.01) ^a^	0.29 (0.21) ^a,b^	0.75 (0.35) ^d^	0.44 (0.30) ^b,c,d^	0.42 (0.35) ^a,b,c,d^	0.09 (0.03) ^a,b^	0.75 (0.23) ^c,d^	0.09 (0.01) ^a,b^	0.52 (0.01) ^c,d^	0.11 (0.01) ^a,b^	3.8 **
(C8:0)	0.29 (0.01) ^a^	1.38 (1.27) ^a,b^	2.73 (1.56) ^b^	1.65 (1.05) ^a,b^	1.18 (0.31) ^a,b^	0.33 (0.19) ^a,b^	1.05 (0.39)^ab^	0.23 (0.01)^a^	1.81 (0.03) ^a,b^	0.30 (0.01) ^a^	3.1 **
(C10:0)	0.26 (0.01) ^a^	0.99 (0.71) ^a^	1.47 (1.08) ^a^	0.66 (0.42) ^a^	0.57 (0.36) ^a^	0.44 (0.20) ^a^	1.09 (0.61)^a^	0.26 (0.01)^a^	0.76 (0.01) ^a^	0.45 (0.01) ^a^	1.5 ns
(C12:0)	0.33 (0.01) ^a^	3.64 (2.92) ^a^	4.42 (3.99) ^a^	2.43 (1.51) ^a^	1.81 (0.53) ^a^	0.85 (0.21) ^a^	2.37 (0.84)^a^	0.74 (0.01)^a^	3.09 (0.04) ^a^	1.29 (0.02) ^a^	1.9 ns
(C14:0)	0.61 (0.01) ^a^	4.12 (5.43) ^a^	5.46 (3.86) ^a^	2.63 (1.16) ^a^	3.71 (2.10) ^a^	0.93 (0.07) ^a^	6.56 (1.83)^a^	0.49 (0.01)^a^	3.31 (0.05) ^a^	0.55 (0.01) ^a^	1.5 ns
(C15:0)	0 (0) ^a^	0.12 (0.16) ^a^	0.24 (0.28) ^a^	0.23 (0.19) ^a^	0 (0) ^a^	0.16 (0.08) ^a^	0.08 (0.03)^a^	0 (0)^a^	0.23 (0.01) ^a^	0 (0) ^a^	1.2 ns
(C16:0)	9.14 (0.13) ^a^	48.96 (50.24) ^a^	149.07 (137.91) ^a^	46.56 (39.24) ^a^	115.20 (25.43) ^a^	13.57 (2.94) ^a^	120.34 (57.35)^a^	12.98 (0.19)^a^	23.85 (0.34) ^a^	12.78 (0.18) ^a^	2.4 ns
(C16:1 [cis-9])	0.59 (0.01) ^a^	1.77 (1.95) ^a^	10.81 (11.80) ^b^	0.68 (0.38) ^a^	1.24 (0.09) ^a^	0.25 (0.10) ^a^	1.62 (0.82)^a^	0.33 (0.01)^a^	0.21 (0.01) ^a^	0.32 (0.01) ^a^	3.5 **
(C17:0)											
(C17:1 [cis-10])	0 (0) ^a^	1.95 (3.87) ^a^	1.60 (1.05) ^a^	2.34 (1.11) ^a^	0 (0) ^a^	5.18 (4.99) ^a^	3.21 (3.06)^a^	0 (0)^a^	2.58 (0.34) ^a^	0 (0) ^a^	0.8 ns
(C18:0)	0 (0) ^a^	0.02 (0.05) ^a^	0 (0) ^a^	1.00 (0.16) ^a^	0 (0) ^a^	1.43 (0.65) ^a^	2.09 (1.22)^a^	0 (0)^a^	0 (0) ^a^	0 (0) ^a^	1.6 ns
(C18:1 [trans-9]) + (C18:1 [cis-9])	10.07 (0.14) ^a,b^	19.58 (9.55) ^a,b^	32.28 (22.78) ^b^	14.76 (7.53) ^a,b^	24.78 (15.43) ^a,b^	7.03 (3.73) ^a^	33.11 (11.29) ^a,b^	14.34 (0.20)^ab^	9.15 (0.13) ^a,b^	13.85 (0.20) ^a,b^	2.6 *
(C18:2 [trans-9,12])	15.84 (0.23) ^a^	21.62 (17.91) ^a^	33.39 (24.03) ^a^	19.59 (12.89) ^a^	35.24 (0.09) ^a^	8.52 (3.58) ^a^	42.93 (13.75)^a^	18.74 (0.27)^a^	10.56 (0.15) ^a^	9.65 (0.14) ^a^	2.3 ns
(C18:2 [cis-9,12])	0 (0) ^a^	2.82 (3.74) ^a^	9.83 (11.35) ^a^	29.06 (18.53) ^a^	0 (0) ^a^	0 (0) ^a^	0 (0)^a^	0 (0)^a^	14.75 (0.21) ^a^	0 (0) ^a^	1.7 ns
(C18:3 [cis-6,9,12])	15.36 (0.22) ^a^	44.26 (35.23) ^a,b^	93.06 (64.14) ^a,b^	46.94 (38.53) ^a,b^	121.73 (14.53) ^b^	23.31 (1.15) ^a^	117.91 (32.98)^ab^	28.00 (0.40)^a^	21.49 (0.31) ^a^	24.08 (0.34) ^a^	5.0 ***
(C20:1 [cis-11])	19.39 (0.28) ^a^	85.87 (74.06) ^a^	283.61 (253.73) ^a^	90.81 (83.28) ^a^	207.81 (21.02) ^a^	35.64 (6.99) ^a^	232.90 (98.51)^a^	34.74 (0.50)^a^	28.13 (0.40) ^a^	36.38 (0.52) ^a^	2.7 ns
(C18:3 [cis-9,12,15])	2.35 (0.03) ^a^	7.33 (3.13) ^a,b,c,d^	7.52 (5.84) ^c,d^	3.83 (1.59) ^a,b,c,d^	2.59 (1.04) ^a,b^	2.39 (0.64) ^a^	10.24 (3.23) ^d^	3.16 (0.05) ^a,b,c^	3.28 (0.05) ^a,b,c^	3.25 (0.05) ^a,b,c^	3.6 **
(C20:3 [cis-11,14,17])	0 (0) ^a^	7.44 (5.68) ^a^	0 (0) ^a^	0 (0) ^a^	0 (0) ^a^	0 (0) ^a^	0 (0) ^a^	0 (0) ^a^	0 (0) ^a^	2.10 (0.01) ^a^	0.8 ns
(C22:2 [cis-13,16])	0 (0) ^a^	0.95 (2.00) ^a^	2.79 (2.22) ^a^	0 (0) ^a^	0 (0) ^a^	5.57 (0.01) ^a^	0 (0)^a^	0 (0)^a^	0 (0) ^a^	0 (0) ^a^	1.9 ns
Total FA content	12.74 (0.18) ^a^	19.27 (12.47) ^a^	106.36 (83.32) ^b^	36.54 (29.33) ^a,b^	92.96 (32.59) ^a,b^	34.21 (4.99) ^a^	18.67 (9.98) ^a,b^	65.01 (0.33) ^a,b^	23.15 (0.12) ^a^	15.61 (0.26) ^a^	3.8 **
MUFA	87.01 (1.24) ^a^	272.35 (200.87) ^a,b^	745.39 (585.93) ^b^	300.15 (221.09) ^a,b^	609.33 (111.84) ^a,b^	238.04 (9.36) ^a,b^	118.79 (35.87) ^a,b^	641.26 (1.96) ^a,b^	137.23 (1.94) ^a,b^	136.66 (1.73) ^a,b^	3.1 *
PUFA	18.78 (0.27) ^a^	30.71 (19.30) ^a^	51.71 (41.68) ^a^	24.10 (6.45) ^a^	39.08 (9.94) ^a^	15.62 (4.31) ^a^	11.17 (6.75) ^a^	54.79 (0.32) ^a^	22.23 (0.20) ^a^	18.10 (0.19) ^a^	2.5 *
UFA	47.49 (0.68) ^a^	160.60 (116.14) ^a,b^	495.65 (386.63) ^b^	203.35 (160.92) ^a,b^	422.49 (67.82) ^a,b^	160.88 (10.88) ^a,b^	77.62 (58.37) ^a,b^	415.82 (1.23) ^a,b^	85.89 (1.10) ^a,b^	81.27 (1.12) ^a,b^	3.3 **
SFA	66.27 (0.95) ^a^	191.31 (135.42) ^a,b^	547.36 (428.31) ^b^	227.45 (167.06) ^a,b^	461.57 (77.76) ^a,b^	176.50 (6.61) ^a,b^	88.78 (73.60) ^a,b^	470.61 (1.54) ^a,b^	108.12 (1.30) ^a,b^	99.37 (1.31) ^a,b^	3.2 **
UFA/SFA	20.73 (0.30) ^a^	81.02 (66.27) ^a^	198.02 (157.62) ^a^	71.70 (54.96) ^a^	147.77 (34.19) ^a^	61.54 (14.13) ^a^	28.58 (7.75)^a^	168.56 (0.42)^a^	29.12 (0.65) ^a^	37.30 (0.42) ^a^	2.7 *
(C6:0)	3.17 (0.05) ^a^	2.51 (0.64) ^a^	2.78 (0.64)	3.22 (0.54) ^a^	3.09 (0.39) ^a^	2.84 (0.14) ^a^	3.87 (0.61) ^a^	3.50 (0.05) ^a^	3.67 (0.03) ^a^	2.82 (0.04) ^a^	1.8 ns

AA—amino acids; FA—fatty acids; MUFA—monounsaturated fatty acids; PUFA—polyunsaturated fatty acids; UFA—unsaturated fatty acids; SFA—saturated fatty acids. ^a–e^ different letters in the same row indicate differences between samples (*p* < 0.05). ns-not significant (*p* > 0.05), *—*p* < 0.05, **—*p* <0.01, ***—*p* < 0.001.

## Data Availability

Data is contained within the article.
